# Erratum: Impact of stem cells in craniofacial regenerative medicine

**DOI:** 10.3389/fphys.2013.00219

**Published:** 2013-08-29

**Authors:** Pedro A. Sanchez-Lara

**Affiliations:** Departments of Pediatrics and Pathology and Laboratory Medicine, Children's Hospital Los Angeles, University of Southern CaliforniaLos Angeles, CA, USA

**Keywords:** stem cell, craniofacial, regeneration

We would like to apply a correction to the authorship and acknowledgments section of this manuscript.

## Authors

Pedro A. Sanchez-Lara^1,2^ and David Warburton^1,2^

^1^Departments of Pediatrics and Pathology and Laboratory Medicine, Keck School of Medicine, Children's Hospital Los Angeles, University of Southern California, Los Angeles, CA, USA

^2^Developmental Biology and Regenerative Medicine Program, Saban Research Institute, Children's Hospital Los Angeles, Los Angeles, CA, USA

